# Wider perioperative glycemic fluctuations increase risk of postoperative atrial fibrillation and ICU length of stay

**DOI:** 10.1371/journal.pone.0198533

**Published:** 2018-06-08

**Authors:** Ming Ann Sim, Weiling Liu, Sophia T. H. Chew, Lian Kah Ti

**Affiliations:** 1 National University Health System, Department of Anaesthesia, Singapore; 2 National University of Singapore, Yong Loo Lin School of Medicine, Singapore; 3 Singapore General Hospital, Department of Anaesthesia, Singapore; Universidade de Mogi das Cruzes, BRAZIL

## Abstract

**Introduction:**

Postoperative atrial fibrillation (POAF) is a common complication following cardiac surgery associated with increased morbidity and mortality. Although sustained hyperglycemia is a known risk factor of AF and poor ICU outcomes, emerging in-vitro studies reveal acute glycemic fluctuations to be an additional independent predictor of AF. The effect of acute glycemic fluctuations on the incidence of POAF in the clinical setting remains unclear. We aim to investigate the effect of the magnitude of acute perioperative glycemic fluctuations on the incidence of POAF in a multi-ethnic Southeast-Asian population.

**Methods:**

We obtained data from1743 patients who underwent elective CABG in a tertiary heart centre from 2009–2011. Patients were kept to a tight baseline glycemic control in accordance with hospital protocol. The magnitude of the difference between the highest and lowest perioperative glucose levels up till the first 48 postoperative hours was employed as a measure of glycemic fluctuation. Patients were divided into 4 groups for analysis based on the magnitude of glycemic fluctuation:A)0-2mmol/L(N = 147); B)>2-4mmol/L(N = 426); C)>4-6mmol/L(N = 513); D)>6mmol/L(N = 657).Our primary outcome was the incidence of POAF. Secondary outcomes included ICU and 30-day mortality and length of stay.

**Results:**

The overall incidence of POAF was 14.7%. This increased as the magnitude of glycemic fluctuation increased, and was statistically highest in Group D(16.4%) as compared with the other 3 sub-groups. Multivariate logistic regression revealed the magnitude of perioperative glycemic fluctuation to be an independent risk factor of POAF(O.R.1.06, 95% C.I.1.01–1.11, p = 0.014).ICU length of stay was statistically highest in Group D(63.1 hours, p = < .001). However, ICU and 30 day mortality rates were similar among the 4 groups.

**Conclusion:**

Increased magnitudes of acute perioperative glycemic fluctuations are associated with a significantly increased risk of POAF and length of ICU stay; and should therefore be minimised but balanced against the risks of hypoglycemia so as to avoid POAF and optimise patient outcomes.

## Introduction

Postoperative atrial fibrillation (POAF) is a common complication following cardiac surgery, occurring in up to 10–60% of cardiac-surgical patients, associated with significantly increased morbidity and mortality rates [1,2].

Sustained hyperglycemia is an established risk factor of atrial fibrillation and adverse ICU outcomes [3,4]. However, in comparison with chronic hyperglycemic states, increased glycemic variability has been shown to be associated with significantly increased oxidative damage as compared to sustained hyperglycemia, and is also known to be associated with adverse ICU outcomes such as mortality and postoperative acute kidney injury [5].

In the chronic setting, apart from the use of the HbA1C (glycosylated hemoglobin) and daily blood sugar monitoring, variability in glucose control has also been proposed as an added measure of adequacy of sugar control due to the increasingly severe oxidative stresses associated with such variations in blood glucose [6]. Studies involving intensive care unit (ICU), cardiac surgical and septic patients have similarly shown glycemic variation to be positively associated with increased mortality and ICU composite outcomes [7–11]. Moreover, emerging in vitro studies have also revealed acute glycemic fluctuations to be an independent predictor of AF [12]. However, the effect of acute glycemic fluctuations on the incidence of POAF in the clinical setting remains unclear, and is unknown in the local multi-ethnic Southeast Asian population undergoing cardiac surgery.

We therefore hypothesize that the magnitude of acute perioperative glycemic fluctuations increases the incidence of postoperative atrial fibrillation. Thus, we aim to investigate the effect of the magnitude of acute perioperative glycemic fluctuation on the incidence of postoperative atrial fibrillation in a multi-ethnic Southeast Asian population undergoing Coronary Artery Bypass Graft surgery (CABG).

## Materials and methods

### Patient selection

Following Sing health Centralised Institutional Review Board approval, we conducted a prospective cohort study of all patients undergoing elective CABG in one Singaporean tertiary heart centre. Data was obtained from 1743 patients receiving elective CABG from January 1 2009 to December 31 2011. Written informed consent was obtained from all patients. Patients were excluded from analysis if they had cardiac arrythmias other than AF postoperatively or if they had missing data regarding postoperative arrythmias.

### Perioperative anaesthesia, surgical and perfusion management

Perioperative surgical management and clinical practices followed international standards. Anesthesia was induced with intravenous induction agents (Etomidate or Propofol) and maintained with a balanced anesthetic regime of low-dose Fentanyl (10–20μg/kg^-1^) and volatile agents (primarily Sevoflourane). Conventional cardiopulmonary bypass circuits with roller pumps, membrane oxygenators, heat exchangers, venous reservoirs, cardiotomy suction and arterial blood filters were used. The volume of prime used in the CPB circuits typically ranged from 1300 to 1400ml. Perfusion targets were mild-to-moderate hypothermia (32–35°C), hematocrit of >22%, activated clotting times of >400s, glucose levels of 10mmol/L, non-pulsatile flow rate of 2.2–2.4L/m^2^ and mean arterial pressure of 50-70mmhg. Myocardial protection was achieved with cold blood cardioplegia. Aprotinin was not used in any patients.

### Perioperative glucose control and monitoring protocol

Patients were kept to a tight baseline blood glucose control of within a range of 4-10mmol/L, in accordance with hospital protocol and the Society of Thoracic Surgeons Practice Guideline series: Blood Glucose Management During Adult Cardiac Surgery [13]. Our hospital protocol aims for a target glucose range from 4-10mmol/L (72mg/dL-180mg/dL), with continuous insulin sliding scale infusions given. In line with hospital protocol, perioperative glycemic targets were strictly adhered to via frequent glucose monitoring every 2–4 hours or more frequently if required, intraoperatively and up to the first 48 hours postoperatively. Due to the frequent monitoring mandated by the protocol, hyperglycemia was promptly recognised and treated. More than 80% of our patients achieved the aforementioned target perioperative glucose levels of within 4-10mmol/L. The highest and lowest intraoperative and postoperative glucose levels (up to the first 48 hours postoperatively) was collected. The magnitude of the difference between the highest and lowest perioperative glucose levels within 48 hours was then calculated as a measure of glycemic fluctuation.

### Magnitude of acute perioperative glycemic fluctuation

Data regarding intra-operative and post-operative glucose levels were collected up till the first 48 hours postoperatively. The magnitude of perioperative glucose fluctuation was defined as the magnitude of the difference between the largest documented perioperative rise or fall in glucose level up till the first 48 hours postoperatively. This was used as a surrogate measure of acute perioperative glycemic variation. Patients were then subdivided into 4 sub-groups for analysis based on the aforementioned calculated magnitude of glycemic variation: Group A) 0-2mmol/L (0–36 mg/dL), Group B) >2-4mmol/L (>36-72mg/dL), Group C) >4-6mmol/L (>72-106mg/dL) and Group D) >6mmol/L (>106mg/dL).

### Incidence of POAF

The primary outcome analysed was the incidence of postoperative atrial fibrillation. A positive incidence of postoperative atrial fibrillation was defined by the development of atrial fibrillation after surgery and before discharge, which lasted more than an hour, and/or affected hemodynamics (systolic blood pressure < 90mmhg or mean arterial pressure < 60mmhg), and/or required electrical and/or pharmacological treatment. All patients received continuous ECG telemetry for at least 72 hours postoperatively, and subsequently received ECGs daily or as and when symptomatic, up to 7 days postoperatively. The diagnosis of POAF was made by clinicians involved in the patient's perioperative management in accordance with institutional protocol.

### Secondary outcomes

ICU length of stay, ICU mortality rate and 30 day mortality rate were analysed as secondary outcomes.

### Statistical analysis

Univariate analysis of population demographics, medical history, pre-operative risk factors, intra-operative variables and post-operative data was carried out. The Chi-square test was used in the analysis of categorical variables. A two-tailed independent samples T-test was used in the analysis of continuous variables. Variables with p<0.05 on univariate analysis were then selected for inclusion in the multivariate logistic regression model.

For comparison of outcomes among the 4 sub-groups of glycemic fluctuation, a one-way ANOVA with post hoc tests was performed for continuous variables.

## Results

We obtained data from a total of 1743 patients who underwent elective CABG at the Singapore General Hospital from 2009–2011. The overall incidence of POAF was 14.7%.

On univariate analysis (Table 1), patients who developed postoperative atrial fibrillation were more likely to have a history of congestive cardiac failure, previous history of atrial fibrillation, increased EuroSCORE, older age, decreased preoperative hemoglobin, and higher preoperative creatinine levels. They were also less likely to be of Indian ethincity and of male gender. Perioperatively, they were more likely to require the use of intra-aortic balloon pumps (IABP) and packed cell transfusions. They were also more likely to have longer bypass times and aortic cross clamp times in addition to lower perioperative hematocrit and postoperative hemoglobin levels. The incidence of postoperative atrial fibrillation significantly increased as the magnitude of glycemic fluctuation increased (Table 1).

**Table 1:** Perioperative patient characteristics and postoperative atrial fibrillation.

	Postoperative Atrial Fibrillation		
Perioperative Patient Characteristics	No POAF /±SD(N = 1487)	POAF/±SD (N = 256)	P value
Age (years)	59.1±10.3	62 .1±9.2	< .001
Body mass index (kg/m^2^)	25.1±4.1	25.0±4.4	.449
Male gender (%)	81.2	73.4	.003
Ethnicity (%)			
Chinese	84.9	15.1	< .001
Malay	80.9	19.1	
Indian	94.7	5.3	
Others	81.7	18.3	
Preoperative creatinine (μmol/L)	104.7±96.1	120.3±132.1	.025
Preoperative dialysis	3.6	5.1	.27
Preoperative Peripheral Vascular Disease	3.8	6.1	.08
Preoperative Cerebrovascular Disease	9.9	8.6	.542
Diabetic (%)	46.8	45.3	.684
Diabetic requiring insulin (%)	7.6	6.3	.519
HbA1c if diabetic (%)	7.5±1.8	7.4±1.7	.690
Preoperative Hemoglobin (g/Dl)	13.6	13.2	.008
Congestive Cardiac Failure (%)	16.8	30.4	< .001
History of Preoperative Atrial Fibrillation (%)	7.5	73.0	< .001
EuroSCORE (Logistic)	3.3	4.9	< .001
Preoperative Left Ventricular Ejection Fraction (%)			
LVEF < 30%	6.6	9.0	0.31
LVEF > 30–40%	22.7	25.4	
LVEF > 40–60%	47.4	45.0	
LVEF > 60%	23.3	22.4	
Need for packed cell transfusion(%)	14.2	21.9	.002
IABP (intra-aortic balloon pump) used (%)	4.5	7.8	.028
Bypass time (mins)	97.9 ±37.1	114.7 ±50.4	< .001
Aortic Cross Clamp time (mins)	56.3±27.4	65.4 ±33.0	< .001
Lowest CPB hematocrit (g/Dl)	24.8 ±4.2	24.1 ±4.1	< .001
ICU first Hemoglobin (g/Dl)	10.6 ±1.8	10.2±1.7	.002
ICU lowest Hemoglobin (g/Dl)	10.0 ±1.4	9.4 ±1.4	< .001
Incidence of hypoglycemia (serum glucose <4mmol/L) (%)	2.0	2.0	.998
Magnitude of perioperative glycemic fluctuation (mmol/l)	5.5±3.0	6.1±3.0	< .001

**Fig 1:**
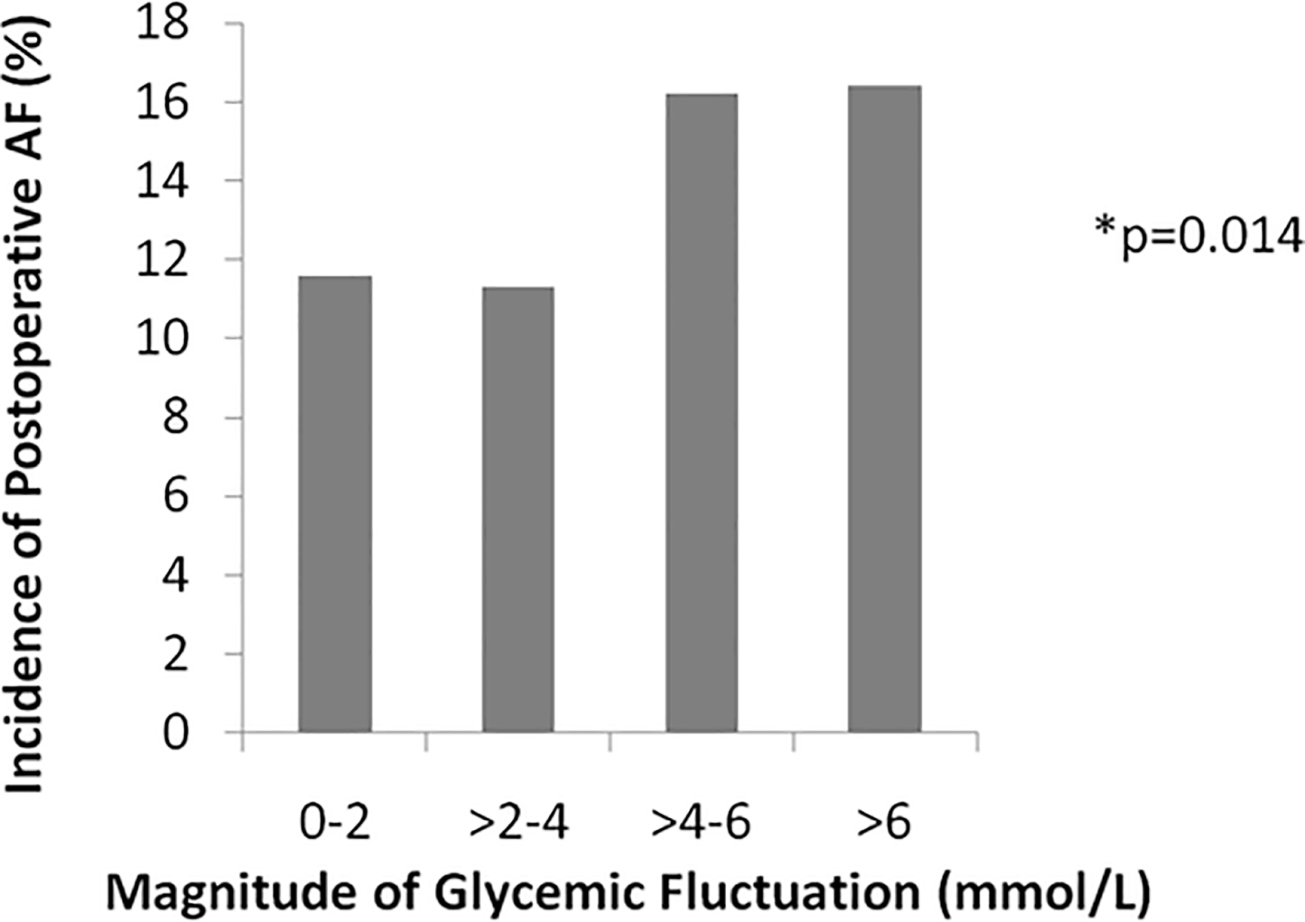
Perioperative glycemic fluctuation and postoperative AF.

Patients were then subdivided into 4 sub-groups for analysis, namely: Group A) 0-2mmol/L, Group B) >2-4mmol/L, Group C) >4-6mmol/L and Group D) >6mmol/L. Upon comparison of the 4 sub groups of glycemic fluctuation, the incidence of POAF was also shown to be significantly highest in Group D (16.4%), followed by Groups C (16.2%), B (11.3%) and A (11.6%) respectively (Fig 1).

Incidence of postoperative atrial fibrillation increases significantly with increased magnitude of perioperative glycemic fluctuation.

Multivariate logistic regression of all significant factors on univariate analysis was performed. Factors which significantly affected the incidence of postoperative atrial fibrillation are presented in Table 2. The magnitude of perioperative glycemic fluctuation was also shown to be an independent risk factor in the development of postoperative atrial fibrillation (O.R. 1.06, 95% C.I. 1.01, 1.11, p = 0.014). In addition to the above, other independent risk factors of POAF also include a previous history of atrial fibrillation, increased age, and longer bypass times. Compared with patients of Chinese ethnicity, patients of Indian ethnicity were also less likely to develop POAF.

**Table 2:** Significant risk factors of postoperative atriai fibrillation on multivariate logistic regression.

Variable	Odds ratio	95% Confidence Interval	P value
History of atrial fibrillation	3.518	2.374, 5.213	< .001
Indian ethnicity (Compared with chinese ethnicity)	0.225	0.097,0.518	< .001
Magnitude of glycemic fluctuation (mmol/L)	1.062	1.012, 1.114	.014
Age (years)	1.020	1.003, 1.037	.020
Bypass time (mins)	1.007	1.002, 1.012	.007
ICU lowest postoperative Hemoglobin (g/Dl)	0.828	0.726, 0.943	.005

Secondary outcomes are described in Table 3. The magnitude of glycemic fluctuation was associated with significantly increased ICU length of stay, but not ICU mortality rate. ICU length of stay was statistically highest (p = < .001) in Group D (63.1hrs) as compared to Groups A (32.7 hrs), B (39.1 hrs) and C (43.5 hrs). However, ICU mortality rate, and 30 day mortality were not significantly different among Groups A, B, C and D (p = 0.118 and 0.23 respectively).

**Table 3:** Secondary outcomes among 4 glycemic sub-groups.

Glycemic Fluctuation Group	A(0-2mmol/L)N = 147	B(>2-4mmol/L)N = 426	C(>4-6mmol/L)N = 513	D(>6mmol/L)N = 657	P value
ICU Length of Stay (hours)	32.7	39.11	43.5	63.1	< .001
ICU Mortality Rate (%)	0.0	0.4	0.5	2.0	.118
Mortality within 30 days (%)	0	0	0.2	0.7	.23

## Discussion

Postoperative atrial fibrillation is a common occurrence in post-cardiac surgery patients, and contributes significantly to mortality and morbidity [14]. Our incidence rate of 14.7% was similar to that reported in previous studies [1].

Our study demonstrates the association between a wider magnitude of glycemic fluctuation and increased incidence of postoperative atrial fibrillation. The stresses associated with CABG predispose patients to an increased risk of stress-induced hyperglycemia [15]. Hyperglycemia is known to increase the risk of development of postoperative atrial fibrillation via the aggravation of interstitial fibrosis and ionic remodeling, which thereby leads to alterations in atrial conduction and structure [16]. Compared with sustained hyperglycemia, wide glycemic fluctuations have been demonstrated to be more specifically associated with mitochondrial superoxide production, inflammatory cytokine release and oxidative damage [12,17,18]. Although these mechanisms are generalised, it is recognised that this increased levels of oxidative stress, cytokine production and inflammation render patients more susceptible to atrial fibrillation via the resultant development of myocardial oxidative injury, cardiac fibrosis and apoptosis [19–21]. We recognise that our study did not investigate the levels of inflammatory markers perioperatively, however, previous studies have similarly proposed that the oscillation in glucose levels is associated with an increase in free radical and cytokine formation, thereby resulting in endothelial dysfunction and oxidative stress on the cellular level, culminating in an inability of cells to compensate adequately via various intracellular defence mechanisms [22]. Our study findings also corroborate with in vitro studies in diabetic mouse models involving wide fluctuating glucose levels ranging from below 5.5mmol/L to greater than 30mmol/L, which also showed an increase in rates of AF with increased glycemic fluctuation [12]. Although our study dealt with far smaller fluctuations in glucose due to our strict glucose control protocol, it is interesting to note that the incidence of POAF was increased even among the smaller fluctuations in glucose, with a near doubling of incidence rate of POAF from 8.8% to 18.6% observed with an increase in glycemic fluctuation of 5mmol/L.

Previous studies have demonstrated diabetic status to be predictive of postoperative atrial fibrillation. However, this was not significantly demonstrated in our study in both univariate (p = 0.684) and multivariate analyses (p = 0.557). It is known that the incidence of metabolic syndrome and insulin insensitivity is higher in the south-east asian population as compared to the western population. Furthermore, due to the high incidence of diabetes in the local south-east asian population, we postulate that our findings also be contributed by the increased prevalence of insulin insensitivity, impaired fasting glucose and pre diabetes in the non-diabetic population.

Other independent predictors of postoperative atrial fibrillation include increased age, preexisting history of atrial fibrillation, increased bypass time, and low postoperative hemoglobin levels. This has been similarly demonstrated in previous work involving the western population [23]. We also demonstrated Indian ethnicity to be associated with a relatively decreased incidence of postoperative atrial fibrillation, this is likely postulated to be due to genetic variations such as a lower prevalence of rs2200733(T) on chromosome 4q25 which is thereby associated with decreased risks of postoperative AF in Indians as compared with the Chinese [24].

Our study also revealed a significant association between a wider magnitude of glycemic fluctuation and increased length of ICU stay. This could likely be explained by the formation of reactive oxygen species, tissue damage, and inflammatory responses (via protein kinase C, polyol pathway, and advanced glycation end products) induced with elevated levels of short term glycemic variability [25]. This also corroborates with previous work by Krinsley et al conducted on general surgical intensive care patients [26]. The difference between the ICU length of stay was almost 24 hours between the highest and lowest groups of glycemic variation. However, our study findings were also contradictory to Krinsley's work, as our mortality rates did not correlate with the magnitude of glycemic fluctuation. This is most likely due to our overall low mortality rates of 1%, which thereby presented challenges in demonstrating a statistically significant trend, and potential variances in heterogeneity between the patient populations.

This study is novel in being the first clinical study to investigate and define the relationship between acute glycemic fluctuations and the risk of postoperative atrial fibrillation in a multi-ethnic Southeast Asian population. It is also a prospectively conducted study of patients undergoing a uniform, quantifiable and predictable stressor of elective CABG, in a controlled perioperative environment with intensive patient monitoring. As compared with previous studies employing the mean, standard deviation and glycemic variability index as a measure of glycemic variability, our use of the maximum perioperative glycemic fluctuation to assess fluctuations in glucose control over time has greater use as a tool and is more intuitive to clinicians [8,9]. This offers greater clinical utility and is easily integrated into daily clinical practice. Moreover, the ranges of glucose fluctuation chosen for sub-analysis in our study were based on two landmark glycemic control protocols—the Society of Thoracic Surgeons Practice Guideline series and Van den Berghe's study which allowed for a maximum glucose fluctuation of 6mmol/L and 2mmol/L respectively [13,27].

Current recommendations by the Society of Thoracic Surgeons Practice Guideline Series allow for a glycemic variation of up to 6mmol/L, however in view of our findings, we postulate that the maximum glycemic variation permitted within these guidelines may still be detrimental, and might not apply to the local south-east Asian population [13]. This therefore underscores the need for more intensive glucose control and minute to minute monitoring protocols in addition to current recommended guidelines.

However, our study may be limited due to the lack of data for continuous glucose monitoring which might have shed greater insight into the effect of minute glycemic fluctuations and glucose variation coefficients on postoperative atrial fibrillation. We have minimised this via the strict adherence to hospital protocol in keeping the glucose control targets within a narrow range of 4-10mmol/L, with more than 80% of patients achieving this target range, and an overall low incidence of hypoglycemia (defined as serum glucose <4mmol/L) of 2%. However, we recognise that a continuous glucose monitoring system would be ideal, as this would track minute glycemic variations, thus functioning as a physiological feedback loop system. Our study may also be limited by the lack of continuous telemetry monitoring postoperatively, which may question the temporal association between POAF and perioperative glycemic variations. However, our findings nonetheless demonstrate an increased incidence of POAF in patients who demonstrated high glycemic fluctuations. However, it is also possible that POAF could increase glycemic variation, where inflammation is a common mechanism [19].

## Conclusions

We conclude that greater perioperative glycemic fluctuations are an independent risk factor for the development of postoperative atrial fibrillation in patients undergoing coronary artery bypass graft surgery.

Despite the strict adherence to baseline glucose control target protocols, large glycemic fluctuations may still occur, hence resulting in an increased risk of developing postoperative atrial fibrillation. Therefore, our findings demonstrate the need for more stringent glucose control and monitoring in addition to the strict baseline glucose control protocols already in place. However, the risk of postoperative atrial fibrillation associated with wide glycemic fluctuations is to also be carefully balanced against the potential risks of hypoglycemia associated with stringent glycemic control. With further technological advancements, we remain optimistic that the advent of continuous glucose monitoring devices may help to avoid even minute fluctuations in glucose, reduce the incidence of perioperative dysglycemia, and eventually contribute to improved patient outcomes.

## Supporting information

S1 File Supporting data file. Deidentified raw data file.
(XLSX)
